# Chronic Bedridden Condition Is Reflected by Substantial Changes in Plasma Inflammatory Profile

**DOI:** 10.3390/biom12121867

**Published:** 2022-12-13

**Authors:** Roberta Magliozzi, Anna Pedrinolla, Stefania Rossi, Anna Maria Stabile, Elisa Danese, Giuseppe Lippi, Federico Schena, Massimiliano Calabrese, Massimo Venturelli Venturelli

**Affiliations:** 1 Department of Neurological and Movement Sciences, Neurology Section, University of Verona, 37100 Verona, Italy; 2 Department of Cellular, Computational, and Integrative Biology, CIBIO, University of Trento, 38123 Trento, Italy; 3 Department of Oncology and Molecular Medicine, Istituto Superiore di Sanità, 00161 Rome, Italy; 4 Department of Surgery and Biomedical Sciences, Section of Human Anatomy, Clinical and Forensic, School of Medicine, 06132 Perugia, Italy; 5 Department of Life and Reproduction Sciences, Laboratory of Clinical Biochemistry, University of Verona, 37134 Verona, Italy; 6 Department of Neuroscience, Biomedicine, and Movement Science, Movement Science Section, University of Verona, 37131 Verona, Italy; 7 Department of Internal Medicine, University of Utah, Salt Lake City, UT 84132, USA

**Keywords:** chronic bedridden condition, plasma biomarkers, inflammatory changes

## Abstract

Absent or reduced physical activity and spontaneous movement over days, weeks, or even years may lead to problems in almost every major organ/system in the human body. In this study, we investigated whether the dysregulation and alteration of plasma protein inflammatory profiling can stratify chronic bedridden conditions observed in 22 elderly chronic bedridden (CBR) individuals with respect to 11 age-matched active (OLD) controls. By using a combination of immune-assay multiplex techniques, a complex of 27 inflammatory mediators was assessed in the plasma collected from the two groups. A specific plasma protein signature is indeed able to distinguish IPO individuals from age-matched OLD controls; while significantly (*p* < 0.001) higher protein levels of IL-2, IL-7, and IL-12p70 were measured in the plasma of CBR with respect to OLD individuals, significantly (*p* < 0.01) higher levels of seven inflammatory mediators, including IL-9, PDGF-b, CCL4 (MIP-1b), CCL5 (RANTES), IL-1Ra, CXCL10 (IP10), and CCL2 (MCP-1), were identified in OLD individuals with respect to CBR individuals. These data suggest that the chronic absence of physical activity may contribute to the dysregulation of a complex molecular pattern occurring with ageing and that specific plasma protein signatures may represent potential biomarkers as well as new potential therapeutic targets for new treatments aimed at improving health expectancy.

## 1. Introduction

It is well known that physical activity, at any age, is crucial for the integrity of functional human physiology, including vascular, cardiac, and respiratory systems but also immune surveillance and the repair capacity. It has been demonstrated that reducing physical activity and spontaneous movement over days, weeks, or even years may lead to problems in almost every major organ [1].

One of the major changes that may occur during ageing is the dysregulation of the immune response, potentially leading to chronic inflammatory conditions. Among the dysregulated proinflammatory mediators, cytokines and chemokines are major culprits in the development of chronic inflammation and the immune-senescence processes [2]. Several studies have demonstrated the increase in serum levels of cytokines, such as TNF and IL-6, in age-related pathogenesis [3,4], with the activation of specific transcription factors possibly associated either with inflammation and/or with alterations of immune system functions. It is important to mention, however, that the type and level of physical activity, diet, and stress may influence these alterations.

With increasing age, there is a physiologic accumulation of cells that have lost their ability to divide and yet do not undergo cell death, termed senescent cells [5]. This process, probably mediated by increased levels of free radicals, may itself be associated with cell-cycle withdrawal, macromolecular issues (DNA, proteins, and lipids), deregulated metabolism, and the increased secretion of a number of pro-inflammatory cytokines, tissue-disrupting matrix metalloproteinases, and growth stimulating factors known as the senescence-associated secretory phenotype (SASP) as well as to the exhaustion of immune response [6,7]. However, all the potential events that may be involved in such alterations accompanying ageing are still not entirely explained. 

A better understanding of the molecular changes and biomarkers related to ageing, changes in physical activity, and senescence are likely to result in the development of novel diagnostic markers as well as potential targets for efficacious future therapies.

In this study, we aimed to verify whether the chronic bed-rest condition may be related to the alteration of physiological inflammatory conditions, as reflected by plasma milieu changes. For this reason, we performed a comprehensive examination of the pattern of inflammatory proteins (cytokines and chemokines) in the plasma of bedridden elderly individuals with respect to age-matched controls.

## 2. Materials and Methods

*Participants.* Twenty-two chronically bedridden individuals (CBR), 80 years old and older, were recruited at the Geriatric Institute Mons. Arrigo Mazzali Foundation (Mantua, Italy). The definition of bedridden was used as when a person cannot get out of bed. Eleven age-matched non-bedridden elderly participants (OLD) were also recruited from the same geographical area (Supplementary Table S1). Non-bedridden participants were able to get out of bed and walk independently. Individuals with evidence of neurodegenerative disease (i.e., Parkinson’s disease or Alzheimer’s disease), heart, liver, or kidney failure, organ transplantation, cerebral haemorrhage, neuromuscular disease, or any other conditions limiting the assessment procedures were excluded from the study in order to avoid alterations due to comorbidities. All experiments were conducted after informed and written consent was obtained from the subjects and their relatives, in accordance with the Declaration of Helsinki, as part of a protocol approved by the Institutional Review Board of the Department of Neurosciences, Biomedicine and Movement Sciences. University of Verona, Italy (Verona, Italy—#CT241123; NIH Clinical trial identification number: NCT03087643).

*Sample collection.* Venous peripheral blood (18 mL) was collected between 9:00 and 10:00 from bedridden individuals and their active counterparts in a fasted state. Within 45 min, plasma was separated from peripheral blood by centrifugation (1200 rpm for 20 min at 4 °C) and kept at −80 °C until analysis. 

*Protein expression level analyses.* The plasma protein levels of a complex pattern of inflammatory mediators were assessed using a multiplex immunoassay platform based on the Luminex technology (Bio-Plex Pro Human Cytokine 27-plex Assay, Bio-Plex X200 System equipped with a magnetic workstation, BioRad, Hercules, CA, USA) previously optimised [8]. To reveal even low concentrations of inflammatory molecules, a specific standard curve was optimised by adding supplementary lower dilution steps. All samples were anonymised and examined blindly with respect to the group condition. Each plasma sample was run in duplicate in the same experiment and in two consecutive experiments to verify the reproducibility and consistency of the results.

*Statistical analysis*. All data are presented as the mean ± SD. All statistical analyses were performed by using GraphPad PRISM (Version 8); to detect differences between the serum protein profiles of the two groups of examined elderly individuals, the non-parametric Mann–Whitney test was used. Pathway analysis was performed by using the Enrich-r analysis available online [9], while the possible types of protein–protein interactions were examined by STRING online software [10]. 

## 3. Results

### 3.1. Subjects Characteristics

The clinical characteristics of the 33 individuals (OLD = 11, CBR = 22) were included and examined in the study. Significant differences between the OLD and IPO cohorts were detected in the following variables: number of comorbidities (*p* = 0.002), cardiovascular disease (*p* = 0.032), number of medications (*p* = 0.039), antipsychotics (*p* < 0.001), antidepressants (*p* < 0.001), and benzodiazepines (*p* = 0.037) (Supplementary Table S1).

### 3.2. Differential Plasma Protein Profiles

**Figure 1:**
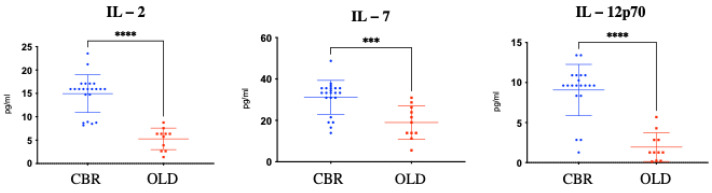
Graphs representing the plasma levels of the molecules found over-expressed in CBR individuals (blue dots) with respect to age-matched OLD control individuals (red squares) as measured by Bio-Plex methodologies. The non-parametric Mann–Whitney test was used for statistical comparison between the two examined groups. *p*-values for each statistically significant comparison have been reported (*** *p* < 0.001; **** *p* < 0.0001).

**Figure 2:**
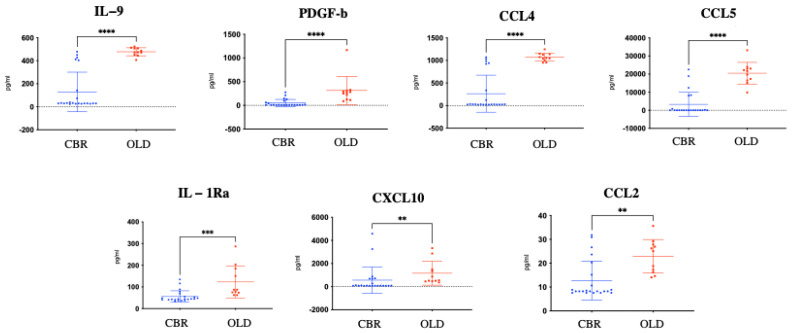
Graphs representing the plasma levels of the molecules found over-expressed in active OLD control individuals (red squares) with respect to CBR individuals (blue dots) as measured by Bio-Plex methodologies. The non-parametric Mann–Whitney test was used for statistical comparison between the two examined groups. *p*-values for each statistically significant comparison have been reported (** *p* < 0.01; *** *p* < 0.001; **** *p* < 0.0001).

When the presence and levels of the 27 examined molecules were analysed in the plasma of the CBR versus the OLD group, significantly (*p* < 0.001) higher protein levels of IL-2, IL-7, and IL-12p70 were measured in the plasma of CBR with respect to the OLD individuals (Figure 1). In contrast, significantly (*p* < 0.01) higher levels of seven inflammatory mediators, including IL-9, PDGF-b, CCL4 (MIP-1b), CCL5 (RANTES), IL-1Ra, CXCL10 (IP10), and CCL2 (MCP-1), were identified in OLD individuals with respect to IPO individuals (Figure 2). 

### 3.3. Pathway Analysis

**Figure 3:**
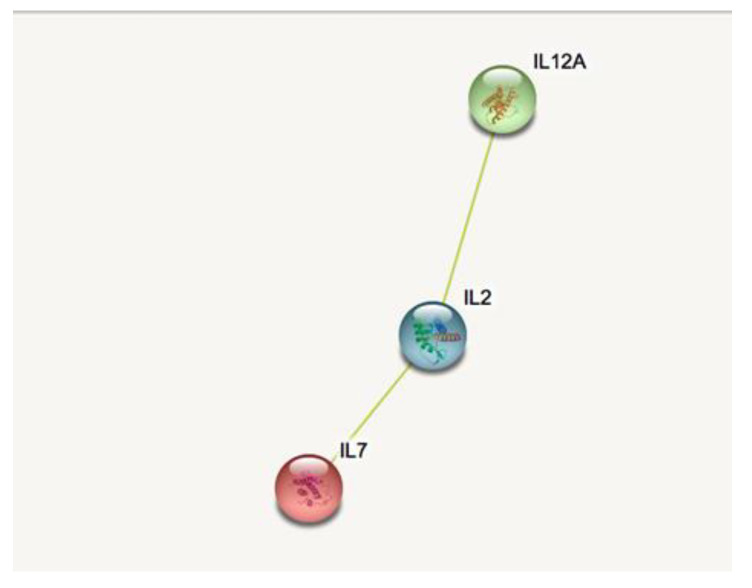
STRING bioinformatic analysis of proteins identified to be over-expressed in bedridden elderly individuals. The protein–protein interaction network was studied and predicted using STRING. The links between the proteins represent possible interactions (line thickness indicates the strength of association and the green line indicates putative text mining associations suggested by further peer-reviewed publications).

**Figure 4:**
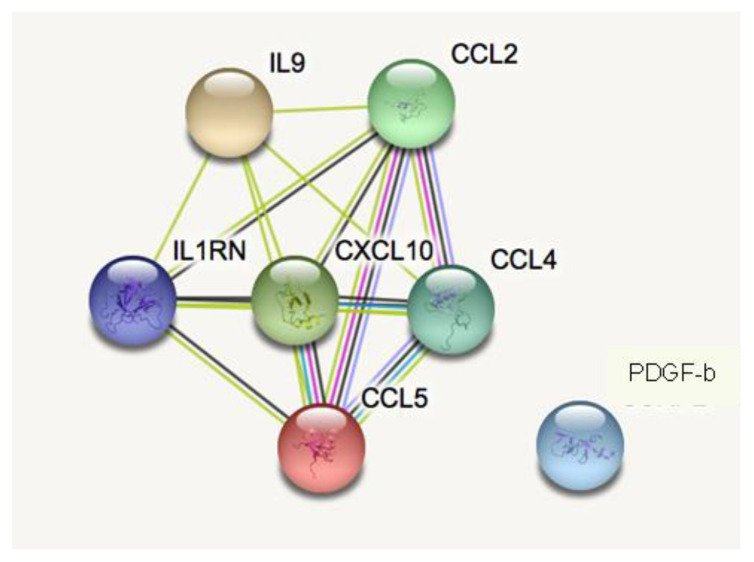
STRING bioinformatic analysis of proteins identified to be over-expressed in age-matched elderly individuals. The non-casual protein–protein interaction network was studied and predicted using STRING. The links between the proteins represent possible interactions (line thickness indicates the strength of association). The green lines indicate putative text mining associations suggested by further peer-reviewed publications; the black lines indicate putative co-expression; the pink lines indicate experimentally determined interactions; the blue lines indicate interactions suggested by curated databases; and the violet lines indicate protein homology.

By performing Enrich-r pathway analyses, the molecules (IL-2, IL-7, and IL-12p70) found over-expressed in the plasma of CBR individuals compared with age-matched OLD individuals, are suggested to be mainly linked to acute T cell activation and immune pro-inflammatory functions (Table 1). Furthermore, the protein–protein interaction analysis STRING validated these results (Supplementary Table S2) and suggested the hypothesis of the possible association among the three inflammatory mediators characterising the plasma of IPO individuals (Figure 3). On the contrary, Enrich-r pathway analysis suggested that the seven molecules found over-expressed in the plasma of OLD individuals are mainly involved in the recruitment and activation of humoral and innate immune responses (PDGF-b, CCL4, CCL5, IL-1Ra, CXCL10, and CCL2) and have a protective anti-inflammatory role, such as IL-9 (Table 2). Furthermore, the identified pathways were validated using a protein–protein interaction analysis STRING (Supplementary Table S3) and indicated strong non-casual putative interactions as well as the potential co-expression at least among six out of the seven inflammatory mediators characterising the plasma of OLD individuals (Figure 4).

## 4. Discussion

New proteomic platforms and methodologies have been recently improved in order to assess the specific protein profile of different pathological conditions from multiple biological matrices, including the plasma, serum, cerebrospinal fluid (CSF), saliva, skeletal muscle, liver, etc. More recently, the use of multiplex protein panels, in particular, has been approved for use in clinical settings, for example, to assess the risk of different cancers, supporting the idea that a set of proteins, rather than a single biomarker, may have clinical utility for the diagnosis, prognosis, and monitoring of several diseases [11]. Several studies have applied these new technologies for the purpose of understanding the potential mechanisms underneath physiological and pathological conditions, while, at the moment, biological ageing and age-related chronic conditions remain not fully explained.

For this reason, in this study, we performed a detailed assessment of the presence and levels of potential inflammatory molecules (cytokines and chemokines) in the plasma of bedridden elderly individuals with respect to age-matched controls, revealing that during ageing, the bedridden condition may significantly modify the plasma protein signature, potentially inhibiting the age-related physiological induction of proteins involved in innate immune regulation and, in contrast, activating pro-inflammatory processes. In fact, we found the decreased expression of a complex protein pattern involved in the recruitment and activation of innate immune functions, such as IL-9, PDGF-b, CCL4, CCL5, IL-1Ra, CXCL10, and CCL2, characterising the plasma protein profile of CBR individuals compared with a group of active, age-matched OLD control counterparts. In addition, a specific pattern of acute, pro-inflammatory molecules (IL-2, IL-7, and IL-12p70) principally linked to T cell activation and inflammatory immune functions, were found over-expressed in the plasma of CBR individuals compared with age-matched controls. These data confirm that chronic physical constraints may induce the differential regulation of inflammatory functions and their corresponding biomarkers.

It was demonstrated that IL-9, a cytokine implicated in human and murine asthma, anaphylaxis, resistance to nematode infection, antiviral immunity, and tumorigenesis, may play a key immunoregulatory role in chronic inflammatory and autoimmune diseases, leading to attenuation of the proinflammatory Th17 response [12]. The lack of its expression in the plasma of CBR individuals with respect to age-matched controls implies that IL-9 may contribute to protection from inflammation, possibly regulating the production of anti-inflammatory cytokines.

At the same time, the over-expression in the plasma of OLD control individuals of several chemokines potentially involved in the recruitment and activation of immune innate response [13] may reflect the tentative physiological regulation of processes of innate immune surveillance and repair activated by ageing. It was suggested that the release of elevated levels of chemokines, such as CCL2, CCL4, CCL5, and CXCL10, involved in the recruitment and activation of B cells, monocytes/macrophages, and dendritic cells in the blood circulation, is physiologically induced by ageing-related changes related to mechanical ventilation or to the vascular and respiratory systems. Such an inflammatory milieu is particularly important to recruit immune cells in sites of local inflammation or infection [13]. Reduced mobility in CBR individuals may affect these physiological repair and regenerative functions and characterise the difficulty or inability to respond to pathological events.

Recent clinical and experimental evidence has suggested that under physiological conditions, the regulation of humoral and complement signalling has a key role in neuronal excitability, synaptic strength, and neurite remodelling promoting nerve regeneration, tissue repair, and healing [14]. On the contrary, in a variety of pathologies, inducing reduced physical activity, as observed in the CBR individuals examined in our study, may lead to the dysregulation of the immune response leading, in turn, to chronic inflammation, persistent pain, and neural dysfunction.

Cell oxidative damage associated with ageing, mainly due to the accumulation of mitochondrial DNA mutations, may induce an increase in reactive oxygen species production and the following development of antioxidant defence systems. This altered system also involves the production of specific inflammatory cytokines/chemokines [15]. It is known that ageing processes, as well as several immunological and neurodegenerative diseases, may alter these capacities. In the current study, we supported this evidence by suggesting the hypothesis that the loss of physical activity may contribute to these alterations.

PDGF-b (together with PDGF-a and their receptors) is known as a potent mitogen and chemoattractant molecule induced by inflammation and oxidative stress. It may play a key role in the alteration of vascular homeostasis during ageing [16]. The PDGF-b over-expression detected in OLD plasma samples with respect to CBR samples could possibly reflect the physiological alteration and remodelling processes of the vascular system due to ageing.

However, this study is not without limitations, as it will need to be validated in an independent and larger population, and possibly, in the future, also compared with a younger population. In addition, longitudinal studies, potentially involving therapeutic approaches, such as increased physical activity, may also propose some molecules as potential biomarkers to monitor ageing conditions.

## 5. Conclusions and Clinical Perspectives

The chronic absence of physical activity may contribute to the dysregulation of complex plasma inflammatory patterns physiologically occurring with ageing. Analysing the presence and levels of 27 inflammatory mediators in the plasma collected from 22 elderly chronic bedridden (CBR) individuals with respect to 11 age-matched active (OLD) controls, we found a specific plasma protein signature able to distinguish the CBR individuals from the age-matched OLD controls; higher protein levels of IL-2, IL-7, and IL-12p70 were measured in the plasma of CBR individuals with respect to OLD individuals, while higher levels of seven inflammatory mediators, including IL-9, PDGF-b, CCL4 (MIP-1b), CCL5 (RANTES), IL-1Ra, CXCL10 (IP10), and CCL2 (MCP-1), were identified in OLD individuals with respect to CBR individuals. These data, even if they need to be validated in larger and independent populations, suggest that specific plasma molecule profiles may propose either novel potential diagnostic biomarkers for patient stratification or new therapeutic targets for new treatments aimed at improving health expectancy.

## Figures and Tables

**Table 1:** Gene ontology analysis of the biological processes possibly linked to the inflammatory mediators (IL-2, IL-7, and IL-12p70) found over-expressed in the plasma of CBR individuals compared with age-matched OLD controls.

Index	Name	*p*-Value	Adjusted *p*-Value	Odds Ratio	Combined Score
1	positive regulation of interleukin-5 production (GO:0032754)	0.002448	0.01958	555.19	3338.15
2	positive regulation of cytokine biosynthetic process (GO:0042108)	0.01392	0.05567	85.27	364.51
3	regulation of cytokine production (GO:0001817)	0.03720	0.09920	30.98	101.95
4	inflammatory response (GO:0006954)	0.08495	0.1699	13.11	32.32
5	positive regulation of cell proliferation (GO:0008284)	0.1393	0.2229	7.71	15.20
6	positive regulation of cellular process (GO:0048522)	0.1681	0.2242	6.27	11.17
7	cytokine-mediated signaling pathway (GO:0019221)	0.2016	0.2304	5.11	8.18
8	regulation of cell proliferation (GO:0042127)	0.2320	0.2320	4.34	6.34

**Table 2:** Gene ontology analysis of the biological processes possibly linked to the inflammatory proteins found over-expressed (PDGF-b, CCL4, CCL5, IL-1Ra, CXCL10, CCL2, and IL-9) in the plasma of OLD control individuals compared with age-matched CBR individuals. The list of the main biological processes related to the complex pattern of molecules is reported according to the combined score.

Index	Name	*p*-Value	Adjusted *p*-Value	Odds Ratio	Combined Score
1	positive regulation of B cell proliferation (GO:0030890)	0.000006519	0.0002255	1426.36	17,031.88
2	regulation of T cell differentiation (GO:0045580)	0.000007912	0.0002255	1288.13	15,131.87
3	negative regulation of B cell apoptotic process (GO:0002903)	0.001050	0.005485	1665.92	11,427.00
4	regulation of B cell proliferation (GO:0030888)	0.00001353	0.0002570	973.46	10,913.37
5	positive regulation of tissue remodeling (GO:0034105)	0.001200	0.005485	1427.86	9603.49
6	bone resorption (GO:0045453)	0.001200	0.005485	1427.86	9603.49
7	negative regulation of lymphocyte apoptotic process (GO:0070229)	0.001349	0.005485	1249.31	8255.55
8	regulation of B cell apoptotic process (GO:0002902)	0.001499	0.005485	1110.44	7220.95
9	interleukin-2-mediated signaling pathway (GO:0038110)	0.001649	0.005485	999.35	6403.33
10	cellular response to interleukin-2 (GO:0071352)	0.001649	0.005485	999.35	6403.33

## Data Availability

All data are available and can be requested from R.M.; most of the data are reported in the main manuscript and Supplementary Materials.

## Supplementary Materials

The following supporting information can be downloaded at: https://www.mdpi.com/article/10.3390/biom12121867/s1, Table S1: Demographic and clinical features of the examined CBR and OLD populations; Table S2: List of the main biological and molecular functions found over-expressed in the plasma of CBR individuals compared with age-matched OLD controls and examined by using STRING protein–protein interaction analysis; Table S3: List of the main biological and molecular functions found over-expressed in the plasma of OLD controls compared with age-matched CBR individuals and examined by using STRING protein–protein interaction analysis.

## References

[1] Walsh K., Roberts J., Bennett G. (1999). Mobility in old age. Gerodontology.

[2] Chung H.Y., Kim D.H., Lee E.K., Chung K.W., Chung S., Lee B., Seo A.Y., Chung J.H., Jung Y.S., Im E. (2019). Redefining chronic inflammation in aging and age-related diseases: Proposal of the senoinflammation concept. Int. Soc. Aging Dis..

[3] Bruunsgaard H., Ladelund S., Pedersen A.N., Schroll M., Jørgensen T., Pedersen B.K. (2003). Predicting death from tumour necrosis factor-alpha and interleukin-6 in 80-year-old people. Clin. Exp. Immunol..

[4] Gordon C.J., Rowsey P.J., Bishop B.L., Ward W.O., MacPhail R.C. (2011). Serum biomarkers of aging in the Brown Norway rat. Exp. Gerontol..

[5] Swenson B.L., Meyer C.F., Bussian T.J., Baker D.J. (2019). Senescence in aging and disorders of the central nervous system. Transl. Med. Aging.

[6] Carreno G., Guiho R., Martinez-Barbera J.P. (2021). Cell senescence in neuropathology: A focus on neurodegeneration and tumours. Neuropathol. Appl. Neurobiol..

[7] Venturelli M., Morgan G.R., Donato A.J., Reese V., Bottura R., Tarperi C., Milanese C., Schena F., Reggiani C., Naro F. (2014). Cellular aging of skeletal muscle: Telomeric and free radical evidence that physical inactivity is responsible and not age. Clin. Sci..

[8] Magliozzi R., Howell O.W., Nicholas R., Cruciani C., Castellaro M., Romualdi C., Rossi S., Pitteri M., Benedetti M.D., Gajofatto A. (2018). Inflammatory intrathecal profiles and cortical damage in multiple sclerosis. Ann. Neurol..

[9] Chen E.Y., Tan C.M., Kou Y., Duan Q., Wang Z., Meirelles G.V., Clark N.R., Ma’Ayan A. (2013). Enrichr: Interactive and collaborative HTML5 gene list enrichment analysis tool. BMC Bioinform..

[10] Magliozzi R., Hametner S., Facchiano F., Marastoni D., Rossi S., Castellaro M., Poli A., Lattanzi F., Visconti A., Nicholas R. (2019). Iron homeostasis, complement, and coagulation cascade as CSF signature of cortical lesions in early multiple sclerosis. Ann. Clin. Transl. Neurol..

[11] Moaddel R., Ubaida-Mohien C., Tanaka T., Lyashkov A., Basisty N., Schilling B., Semba R.D., Franceschi C., Gorospe M., Ferrucci L. (2021). Proteomics in aging research: A roadmap to clinical, translational research. Aging Cell.

[12] Ruocco G., Rossi S., Motta C., Macchiarulo G., Barbieri F., De Bardi M., Borsellino G., Finardi A., Grasso M.G., Ruggieri S. (2015). T helper 9 cells induced by plasmacytoid dendritic cells regulate interleukin-17 in multiple sclerosis. Clin. Sci..

[13] Valentine M.S., Link P.A., Herbert J.A., Kamga Gninzeko F.J., Schneck M.B., Shankar K., Nkwocha J., Reynolds A.M., Heise R.L. (2018). Inflammation and Monocyte Recruitment Due to Aging and Mechanical Stretch in Alveolar Epithelium are Inhibited by the Molecular Chaperone 4-Phenylbutyrate. Cell. Mol. Bioeng..

[14] Warwick C.A., Keyes A.L., Woodruff T.M., Usachev Y.M. (2021). The complement cascade in the regulation of neuroinflammation, nociceptive sensitization, and pain. J. Biol. Chem..

[15] Camougrand N., Rigoulet M. (2001). Aging and oxidative stress: Studies of some genes involved both in aging and in response to oxidative stress. Respir. Physiol..

[16] Ouyang L., Zhang K., Chen J., Wang J., Huang H. (2018). Roles of platelet-derived growth factor in vascular calcification. J. Cell. Physiol..

