# Supplementary material for: Chronic Bedridden Condition Is Reflected by Substantial Changes in Plasma Inflammatory Profile
Source: Biomolecules. 2022 Dec 13;12(12):1867. doi: 10.3390/biom12121867 (PMC9775060; doi:10.3390/biom12121867)
Supplement: Supplementary file 1 [file biomolecules-12-01867-s001.zip › biomolecules-2102114-supplementary.pdf]

Supplementary Table S1

Characteristic	OLD (n = 11)	CBR (n = 22)
Age - years	85 ± 7	88 ± 6
Female - n (%)	6 (70)	13 (55)*
Bedridden - years	0	3.8 ± 2.3*
Weight - kg	65 ± 12	61 ± 15
Height - m	1.6 ± 0.4	1.6 ± 0.6
BMI - $kg \cdot m^{-2}$	24.3 ± 4.3	25.2 ± 6.0
Comorbidities		
Number of comorbidities per individual	3 ± 1	4 ± 1
CardiovascularDisease - n (%)	2 (6)	4 (12)
Diabetes - n (%)	4 (12)	3 (9)
Arthrosis- n (%)	3 (9)	4 (12)
Pharmacological Treatment		
Number of medications per individual	1 ± 0.5	4.2 ± 2.2*
Antihypertensive - n (%)	4 (12)	8 (24)*
Cardiovascularmedication– n (%)	2 (6)	3 (9)
Antipsychotics and antidepressant - n (%)	0	11 (32)*
Benzodiazepines – n (%)	2 (6)	7 (20)*

Supplementary Table S1. Demographic and clinical features of the examined CBR and OLD populations.*= significant difference between groups, $p<0.05$

Supplementary Table S2

Functional enrichments in your network

explain columns

Biological Process (Gene Ontology)

GO-term	description	count in network	strength	false discovery rate
GO:0045582	positive regulation of T cell differentiation	3 of 78	2.4	3.65e-05
GO:0050671	positive regulation of lymphocyte proliferation	3 of 130	2.18	5.46e-05
GO:0042110	T cell activation	3 of 225	1.94	5.56e-05
GO:0032660	regulation of interleukin-17 production	2 of 23	2.75	0.00011
GO:0001819	positive regulation of cytokine production	3 of 390	1.7	0.00016
GO:0030890	positive regulation of B cell proliferation	2 of 42	2.49	0.00025
GO:0010469	regulation of signaling receptor activity	3 of 577	1.53	0.00042
GO:0042531	positive regulation of tyrosine phosphorylation of STAT prot...	2 of 60	2.34	0.00046
GO:0032729	positive regulation of interferon-gamma production	2 of 66	2.3	0.00050
GO:0019221	cytokine-mediated signaling pathway	3 of 655	1.48	0.00052
GO:0046634	regulation of alpha-beta T cell activation	2 of 86	2.18	0.00073
GO:0002708	positive regulation of lymphocyte mediated immunity	2 of 88	2.17	0.00074
GO:0002824	positive regulation of adaptive immune response based on ...	2 of 89	2.17	0.00074
GO:0042102	positive regulation of T cell proliferation	2 of 92	2.15	0.00078
GO:0097191	extrinsic apoptotic signaling pathway	2 of 93	2.15	0.00078
GO:0030217	T cell differentiation	2 of 131	2.0	0.0013
GO:0009967	positive regulation of signal transduction	3 of 1493	1.12	0.0032
GO:0042981	regulation of apoptotic process	3 of 1501	1.12	0.0032
GO:0045927	positive regulation of growth	2 of 252	1.71	0.0035
GO:0006955	immune response	3 of 1560	1.1	0.0035
GO:0002250	adaptive immune response	2 of 280	1.67	0.0039
GO:0031349	positive regulation of defense response	2 of 365	1.55	0.0062
GO:0032103	positive regulation of response to external stimulus	2 of 499	1.42	0.0107
GO:0008285	negative regulation of cell population proliferation	2 of 669	1.29	0.0175
GO:0043066	negative regulation of apoptotic process	2 of 859	1.18	0.0271
GO:0002252	immune effector process	2 of 927	1.15	0.0304
GO:1902533	positive regulation of intracellular signal transduction	2 of 959	1.13	0.0315
GO:0007267	cell-cell signaling	2 of 1073	1.08	0.0372
GO:0051241	negative regulation of multicellular organismal process	2 of 1098	1.07	0.0383
GO:0048523	negative regulation of cellular process	3 of 4454	0.64	0.0485

Molecular Function (Gene Ontology)

GO-term	description	count in network	strength	false discovery rate
GO:0005125	cytokine activity	6 of 216	1.89	9.38e-10
GO:0005126	cytokine receptor binding	6 of 272	1.79	1.83e-09
GO:0008009	chemokine activity	4 of 48	2.37	2.67e-08
GO:0048020	CCR chemokine receptor binding	3 of 41	2.31	3.58e-06
GO:0031726	CCR1 chemokine receptor binding	2 of 6	2.97	2.09e-05
GO:0031730	CCR5 chemokine receptor binding	2 of 7	2.9	2.39e-05
GO:0048019	receptor antagonist activity	2 of 14	2.6	6.87e-05
GO:0070851	growth factor receptor binding	2 of 131	1.63	0.0043
GO:0019887	protein kinase regulator activity	2 of 193	1.46	0.0085

Supplementary Table S2. List of the main biological and molecular functions found over-expressed in the plasma of CBR individuals compared with age-matched OLD controls and examined by using STRING protein-protein interaction analysis.

Supplementary Table S3

Functional enrichments in your network

explain columns

GO-term	description	count in network	strength	false discovery rate
**Biological Process (Gene Ontology)**				
GO:0045582	positive regulation of T cell differentiation	3 of 78	2.4	3.65e-05
GO:0050671	positive regulation of lymphocyte proliferation	3 of 130	2.18	5.46e-05
GO:0042110	T cell activation	3 of 225	1.94	5.56e-05
GO:0032660	regulation of interleukin-17 production	2 of 23	2.75	0.00011
GO:0001819	positive regulation of cytokine production	3 of 390	1.7	0.00016
GO:0030890	positive regulation of B cell proliferation	2 of 42	2.49	0.00025
GO:0010469	regulation of signaling receptor activity	3 of 577	1.53	0.00042
GO:0042531	positive regulation of tyrosine phosphorylation of STAT prot...	2 of 60	2.34	0.00046
GO:0032729	positive regulation of interferon-gamma production	2 of 66	2.3	0.00050
GO:0019221	cytokine-mediated signaling pathway	3 of 655	1.48	0.00052
GO:0046634	regulation of alpha-beta T cell activation	2 of 86	2.18	0.00073
GO:0002708	positive regulation of lymphocyte mediated immunity	2 of 88	2.17	0.00074
GO:0002824	positive regulation of adaptive immune response based on ...	2 of 89	2.17	0.00074
GO:0042102	positive regulation of T cell proliferation	2 of 92	2.15	0.00078
GO:0097191	extrinsic apoptotic signaling pathway	2 of 93	2.15	0.00078
GO:0030217	T cell differentiation	2 of 131	2.0	0.0013
GO:0009967	positive regulation of signal transduction	3 of 1493	1.12	0.0032
GO:0042981	regulation of apoptotic process	3 of 1501	1.12	0.0032
GO:0045927	positive regulation of growth	2 of 252	1.71	0.0035
GO:0006955	immune response	3 of 1560	1.1	0.0035
GO:0002250	adaptive immune response	2 of 280	1.67	0.0039
GO:0031349	positive regulation of defense response	2 of 365	1.55	0.0062
GO:0032103	positive regulation of response to external stimulus	2 of 499	1.42	0.0107
GO:0008285	negative regulation of cell population proliferation	2 of 669	1.29	0.0175
GO:0043066	negative regulation of apoptotic process	2 of 859	1.18	0.0271
GO:0002252	immune effector process	2 of 927	1.15	0.0304
GO:1902533	positive regulation of intracellular signal transduction	2 of 959	1.13	0.0315
GO:0007267	cell-cell signaling	2 of 1073	1.08	0.0372
GO:0051241	negative regulation of multicellular organismal process	2 of 1098	1.07	0.0383
GO:0048523	negative regulation of cellular process	3 of 4454	0.64	0.0485

GO-term	description	count in network	strength	false discovery rate
**Molecular Function (Gene Ontology)**				
GO:0005126	cytokine receptor binding	3 of 272	1.86	2.47e-05
GO:0005125	cytokine activity	3 of 216	1.96	1.66e-05
GO:0070851	growth factor receptor binding	3 of 131	2.17	1.13e-05
GO:0008083	growth factor activity	3 of 160	2.09	1.13e-05

Supplementary Table S3. List of the main biological and molecular functions found over-expressed in the plasma of OLD controls compared with age-matched CBR individuals and examined by using STRING protein-protein interaction analysis.

Supplementary Table S4

**Targets**	**Assay Working Range (pg/ml)**<br>**LLOQ - ULOQ**	**Assay Precision**<br>**%CV**
IL-1ra	4.46 - 19409.64	2.5
IL-2	1.36 - 1877.62	1.36
IL-7	0.5 - 9517.42	2.4
IL-9	0.68 - 44521.16	2.3
IL-12p70	0.32 - 20257.68	1.7
PDGF-b	0.4 - 6778.53	3.5
CL2 (MCP-1)	0.4 - 1856.64	2.2
CCL4 (MIP-1b)	3.92 - 72682.14	2.0
CCL5 (RANTES)	0.95 - 11771.91	2.8
CXCL10(IP-10)	3.34 - 50375.34	2.8

Supplementary Table S4. Performance caracteristics of protein expression levels analysis by Bio-Plex assay.
